# Soft tissue dimensional changes after alveolar ridge preservation using different sealing materials: a systematic review and network meta-analysis

**DOI:** 10.1007/s00784-021-04192-0

**Published:** 2021-10-20

**Authors:** Luigi Canullo, Paolo Pesce, Donato Antonacci, Andrea Ravidà, Matthew Galli, Shahnawaz Khijmatgar, Grazia Tommasato, Anton Sculean, Massimo Del Fabbro

**Affiliations:** 1 Department of Periodontology, University of Bern, Bern, Switzerland; 2 Via Nizza, 46, 00198 Rome, Italy; 3 Department of Surgical Sciences, University of Genova, Genova, Italy; 4 Private Practice, Bari, Italy; 5 Department of Periodontics and Oral Medicine, School of Dentistry, University of Michigan, Ann Arbor, MI USA; 6 Department of Biomedical, Surgical and Dental Sciences, Università Degli Studi Di Milano, Milan, Italy; 7 IRCCS Orthopedic Institute Galeazzi, Milan, Italy

**Keywords:** Collagen membrane, Non-crosslinked, Crosslinked, Collagen sponge, Network meta-analysis, Multidimensional scale, Ranking, SUCRA, Predictive interval, Soft tissue, Alveolar ridge preservation

## Abstract

**Background:**

Alveolar ridge preservation (ARP) is a proactive treatment option aiming at attenuating post-extraction hard and soft tissue dimensional changes. A high number of different types of biomaterials have been utilized during ARP to seal the socket, but their effectiveness in terms of soft tissue outcomes has rarely been investigated and compared in the literature.

**Objective:**

To evaluate the efficacy of different types of membranes and graft materials in terms of soft tissue outcomes (keratinized tissue width changes, vertical buccal height, and horizontal changes) after ARP, and to assign relative rankings based on their performance.

**Materials and methods:**

The manuscript represents the proceedings of a consensus conference of the Italian Society of Osseointegration (IAO).

PUBMED (Medline), SCOPUS, Embase, and Cochrane Oral Health’s Information Specialist were utilized to conduct the search up to 06 April 2021. English language restrictions were placed and no limitations were set on publication date. Randomized controlled trials that report ARP procedures using different sealing materials, assessing soft tissue as a primary or secondary outcome, with at least 6-week follow‐up were included.

Network meta-analysis (NMA) was performed using mean, standard deviation, sample size, bias, and follow-up duration for all included studies. Network geometry, contribution plots, inconsistency plots, predictive and confidence interval plots, SUCRA (surface under the cumulative ranking curve) rankings, and multidimensional (MDS) ranking plots were constructed.

**Results:**

A total of 11 studies were included for NMA. Overall, the level of bias for included studies was moderate. Crosslinked collagen membranes (SUCRA rank 81.8%) performed best in vertical buccal height (VBH), autogenous soft tissue grafts (SUCRA rank 89.1%) in horizontal width change (HWch), and control (SUCRA rank 85.8%) in keratinized mucosa thickness (KMT).

**Conclusions:**

NMA confirmed that the use of crosslinked collagen membranes and autogenous soft tissue grafts represented the best choices for sealing sockets during ARP in terms of minimizing post-extraction soft tissue dimensional shrinkage.

**Clinical relevance:**

Grafting materials demonstrated statistically significantly better performances in terms of soft tissue thickness and vertical buccal height changes, when covered with crosslinked collagen membranes. Instead, soft tissue grafts performed better in horizontal width changes. Non-crosslinked membranes and other materials or combinations presented slightly inferior outcomes.

## Introduction

Post-extraction hard and soft tissue dimensional changes are an inevitable biologic process [1–6] that must be accounted for during dental implant site development [7, 8]. Several studies have described the healing process after extractions both in animals and humans, providing a better understanding of post-extraction soft and hard tissue remodelling from a histologic perspective [4, 5]. While bone remodelling is relatively well understood, a more thorough understanding of post-extraction soft tissue changes is required [9]. Thicker soft tissues have been shown to respond favorably after periodontal or implant surgery in terms of wound healing [10, 11]. A recent NMA supports the view that thick supracrestal tissue can provide significantly less marginal bone loss [12]. A subsequent clinical trial by Garaicoa-Pazmino found that by providing more space for the formation of the supracrestal gingiva through tissue level implants, the marginal bone loss difference between gingival phenotypes could be mitigated to the level of non-significance at 1-year follow-up [13]. However, the influence of various alveolar ridge preservation (ARP) techniques on soft tissue outcomes remains to be determined [3].

The underlying molecular and cellular mechanisms regulating new bone formation also play a large role in governing soft tissue extracellular matrix remodelling [14, 15]. During post-extraction healing, soft tissue thickens while the bone is gradually resorbed [3]. Although one possible benefit of this process is that soft tissue thickness tends to increase, post-extraction soft tissue changes may potentially mask the true extent of alveolar ridge atrophy [16, 17].

Ultimately, ARP does not prevent post-extraction ridge atrophy from occurring, but may limit the extent to which it occurs [18]. Interestingly, several studies have shown a reduction in keratinized soft tissue after tooth extraction [3, 19], underlining the potential need to perform additional soft tissue augmentation procedures for implant site development [20]. Chappuis et al. report in their literature review that no significant differences between the biomaterials and techniques used for ARP were found; however, the types of treatments and biomaterials have not been separated for bone filling and socket sealing, so further investigation is needed to clarify these aspects [17]. Although osseous post-extraction changes are relatively well-characterized, soft tissue dimensional changes using different biomaterials are less well understood. Hence, the present systematic review aimed to evaluate and compare the effects of different ARP techniques on post-extraction soft tissue dimensions. In addition, a network meta-analysis (NMA) was performed, to rank which sealant material used in ARP procedures achieved the best results.

## Materials and methods

The present review was conducted according to PRISMA guidelines (http://www.prisma-statement.org/) and the protocol was registered with PROSPERO (CRD42020218153).

The manuscript represents the proceedings of a consensus conference of the Italian Society of Osseointegration (IAO, https://www.iao-online.com).

The focused questions were elaborated following the PICOT format:

- Patients (P)—patients undergoing tooth extraction with or without ARP.
- Intervention (I)—ARP using different bone grafts (autogenous bone “AU,” bone marrow aspirates “MA,” xenografts “XG,” allografts “AG,” alloplastic grafts “AP,” autogenous tooth grafts “ATG,” as well as bioactive agents (including autologous platelet concentrates, recombinant growth factors, and statins) “BIO”), and membrane biomaterials (resorbable crosslinked collagen membranes “CM-Cross,” resorbable non-crosslinked collagen membranes “CM-NonCross,” resorbable synthetic membranes “Resorb:Syn,” autogenous soft tissue grafts “Auto,” collagen sponges “ColS,” non-resorbable membranes).
- Comparison (C)—all possible comparisons among the included interventions were explored, including spontaneous healing.
- Outcome (O)—for soft tissues, the following outcomes were evaluated and compared: horizontal width linear changes (mm), vertical buccal linear changes (mm), keratinized mucosa thickness (KMT) changes (mm).
- Time (T)—at least 6-week follow-up after extraction.

### Focused questions

The focused questions leading the review process were the following:

1. What ARP biomaterials produced the most beneficial effects compared spontaneous healing in terms of KMT as well as horizontal and vertical dimensional soft tissue changes?
2. What ARP biomaterial was associated with the lowest three-dimensional soft tissue changes post-extraction compared to other materials?

### Eligibility criteria

Randomized controlled trials (RCTs) focused on post-extraction ARP techniques with either parallel or split-mouth designs, treating at least 10 patients (at least 5 patients per group), and evaluating soft tissue changes in either the horizontal or vertical dimensions with at least 6-week follow-up post-extraction, were included. Studies had to present data in the form of mean and standard deviation for at least one of the following parameters to be included: horizontal width linear changes (mm), vertical (buccal and/or lingual/palatal and/or midline height) linear changes (mm), volumetric (3-dimensional) changes (mm^3^), KMT changes (mm). If none of the above variables were provided, or mean and standard deviation were unavailable, the study was excluded. In case of studies with multiple test and/or control groups, only the groups pertinent to the present review were included in analyses.

### Search strategy

A literature search was conducted through electronic databases (MEDLINE (PubMed), EMBASE, Cochrane Central Register of Controlled Trials, and Scopus) using an ad-hoc search string that was adapted to each database: (((((((“tooth extraction”) OR “socket”) OR “alveolus”) OR “dental extraction”)) AND ((((((((((“bone grafts”) OR “biomaterials”) OR “autografts”) OR “collagen”) OR “cell therapy”) OR “platelet concentrates”) OR “alloplasts”) OR “allografts”) OR “xenograft”) OR “bioceramic scaffolds”))) AND (((((“alveolar ridge preservation”) OR “socket preservation”) OR “socket grafting”) OR “socket filling”) OR “ridge maintenance”) AND ((“soft tissue OR “mucosa”) AND ((“horizontal width” OR (“vertical” OR “buccal” OR “vestibular “ OR “lingual” OR “palatal” OR “volume”) AND “change*”). The last electronic search was carried out on 06 April 2021. A manual search was also performed through the following journals: *British Dental Journal*, *British Journal of Oral and Maxillofacial Surgery*, *Clinical Implant Dentistry and Related Research*, *Clinical Oral Implants Research*, *Clinical Oral Investigations*, *International Journal of Oral Implantology*, *European Journal of Oral Implantology*, *European Journal of Oral Sciences*, *Implant Dentistry*, *International Journal of Oral and Maxillofacial Implants*, *International Journal of Oral and Maxillofacial Surgery*, *International Journal of Periodontics and Restorative Dentistry*, *Journal of Clinical Periodontology*, *Journal of Dental Research*, *Journal of Dentistry*, *Journal of Maxillofacial & Oral Surgery*, *Journal of Oral and Maxillofacial Surgery*, *Journal of Periodontal Research, Journal of Periodontology*, *Oral Surgery*, *Oral Medicine*, *Oral Pathology*, and *Oral Radiology and Endodontology*.

The reference lists of identified RCTs and also relevant systematic reviews were scanned for possible additional studies. Online registries providing information about in-progress clinical trials were reviewed (http://clinicaltrials.gov/; http://www.centerwatch.com/clinicaltrials/; http:// www.clinicalconnection.com/). English language restrictions were placed and no limitations were set on publication dates.

### Study selection

Two authors (SK and DA) independently selected the relevant articles. After the first screening based on abstract and titles, a list of eligible studies was set. The full text was retrieved for each eligible study, and was examined to check if the studies met the inclusion and exclusion criteria, as well as to extract data for qualitative and quantitative analysis, and for risk of bias assessment. For the study selection process, any differences in opinions and agreement in including the articles were discussed with logical reasoning, and when the agreement was not met, a third author (MDF) was consulted to make a decision and finalize the list of included studies. Interrater reliability (IRR) was assessed to identify the extent to which two reviewers interpreted the data in the same way (concordance) and assigned the same code. In order to quantify the IRR, Cohen’s k statistic was conducted and interpreted as ≤ 0 (indicating no agreement), 0.01–0.20 (none to slight), 0.21–0.40 (fair), 0.41–0.60 (moderate), 0.61–0.80 (substantial), and 0.81–1.00 (almost perfect agreement). A score of ≥ 80% was considered adequate result to satisfy the IRR.

### Data collection

Relevant data (e.g., study design, number of surgical sites, antibiotic prescription, presence/absence of buccal wall, primary/secondary intention healing, smoking habits, and intra-/post-operative complications) were retrieved from included studies and collected in a predetermined datasheet for subsequent analysis. The main study outcomes were the following:

- Changes in KMT measured clinically with a probe, ultrasonic gingival meter, or digitally through STL file (intraoral scanning or desktop scanning of models) superimposition with DICOM files from cone beam computed tomography (CBCT).
- Changes in vertical buccal and palatal/lingual soft tissue height measured clinically with a probe/stent, digitally through STL file superimposition.
- Horizontal width changes measured clinically with a probe or digitally through STL file superimposition at different vertical distances from the crest. Measurements taken at different vertical distances were averaged to enable NMA.

### Risk of bias assessment

Two reviewers (SK and DA) performed the risk of bias assessment independently. Disagreements were resolved by consulting with a third author (MDF). Risk of bias of the included trials was assessed based on the following criteria: randomization method, concealed allocation of treatment, blinding of outcome assessors, completeness of outcome assessment reporting, and completeness of information on reasons for withdrawal by the trial group. All such criteria were scored as adequate/non-adequate/unclear. The performance bias domain was not evaluated, because in ARP procedures, the technique used is impossible to conceal from both the clinician and the patient, especially in spontaneous healing groups. Studies were classified as low risk of bias (plausible bias unlikely to seriously alter the results) if all criteria were judged adequate; moderate risk of bias (plausible bias that raises some doubt about the results) if one or more criteria were considered unclear and none were inadequate; or high risk of bias (plausible bias that seriously weakens confidence in the results) if one or more criteria were judged inadequate. The criteria for assessing the risk of bias of RCTs were adapted from the tool reported in the Cochrane Handbook for Systematic Reviews of Interventions [21]. The risk of bias in the different studies affects the reliability of the comparisons reported, in the Network Geometry Plot of each outcome, by coloring the edges: green (high reliability), yellow (moderate reliability), and red (low reliability).

### Data analysis

The number of studies selected for NMA was based on the different sealing materials used in each test and control group. Each included study compared at least two different ARP sealing materials (control/spontaneous healing, autogenous grafts, resorbable crosslinked collagen membranes, resorbable non-crosslinked collagen membranes, collagen sponges, and resorbable synthetic membranes). The mean difference, standard deviation (SD), type of treatment, and number of subjects involved were collected for further analysis. Soft tissue dimensional changes (vertical buccal height, KMT, and horizontal width changes) were collected. In situations where two different materials were compared in only a single study, the comparison was excluded as there would be network disconnection. Data retrieved from the included studies were used to generate network geometry plots in order to compare treatment interventions. Contribution plots, inconsistency plots, predictive interval plots, surface under the cumulative ranking curve (SUCRA), and multidimensional scale rankings were used to present the results of the NMA. The NMA was reported in accordance with Hutton et al. 2015 [22]. The strength of the evidence was assessed using the GRADE criteria for NMA [23]. The direct, indirect, and NMA evidence was calculated using node splitting methods. The NMA was carried out using meta and mvmeta network commands in conjunction with STATA software (STATA/IC 16.1, StataCorp LLC, 4905 Lakeway Drive College Station, TX, 77,845, USA). To obtain data feasible for NMA, the following variables were considered: study id, author, treatment (*t*), mean, SD, number of subjects in test and control groups (*n*), and blinding to assess the risk of bias (1, low risk; 2, moderate risk; 3, high risk). Furthermore, to avoid network disconnections, calcium sulfate barriers [24], PLA membranes, and PLGA membranes were aggregated and categorized as synthetic resorbable materials, whereas soft cortical porcine laminae [25] was considered a crosslinked membrane. The effect estimates were calculated and illustrated in the Prl and Crl plots.

## Results

**Fig. 1:**
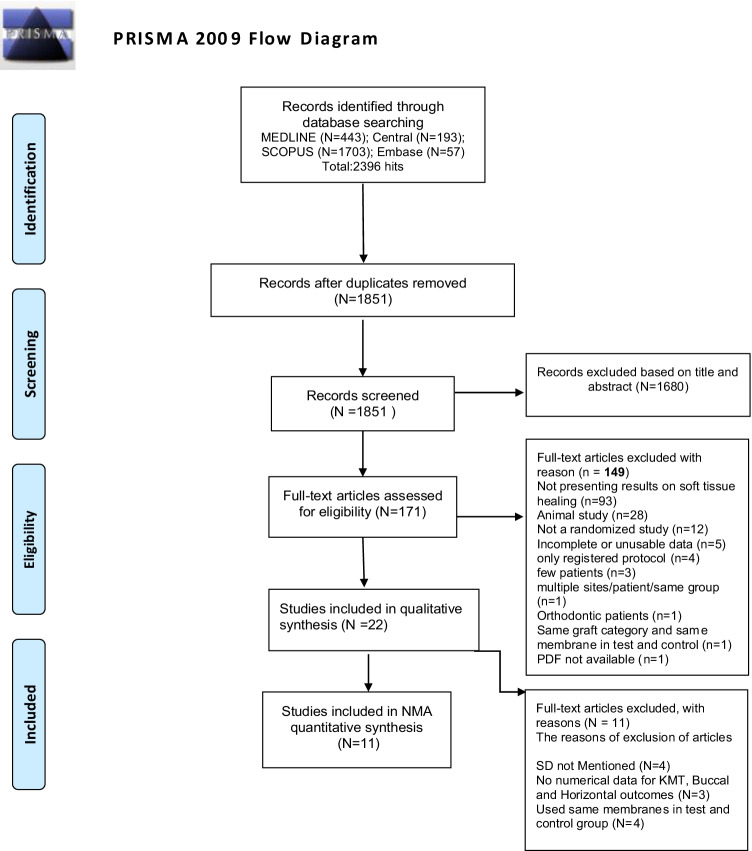
Flowchart of the study selection process

The research strategy initially identified 2,396 potential articles, of which 1,680 were excluded based on title and abstract screening, and 149 were subsequently excluded according to the aforementioned eligibility criteria (Fig. 1). The most frequent reasons for exclusion were the absence of reported outcomes concerning soft tissue dimensional changes, followed by study design (animal studies were excluded). The IRR score from Cohen’s k statistic at the full text article selection stage was 0.81 (81%), suggestive of substantial agreement between the reviewers. Overall, 22 articles were included in the qualitative synthesis [18, 24–44]. All included studies were RCTs and are described in detail in Table 1 and Table 2. Of these, four were excluded from quantitative analyses because they did not report SD, three were excluded because they did not report numerical data, and three others were excluded because they used the same membrane in both the test and control groups. Ultimately, 11 articles were included in the quantitative NMA (Table 3) [24–26, 29, 31, 33, 38–41, 43]. Seventeen studies had a parallel design, and five had a split-mouth design. Medication prescription was reported in 16 studies, and 10 of these administered post-operative antibiotics.

**Table 1:** Characteristics of the study included

Author Year	Type of RCT	Country	Smoker	Setting	Private sponsor	Antibiotics	Buccal wall	Subject	Healing 1°/2°	Test filler	Control filler	Test sealing	Control sealing	Outcome variables	Measuring technique
*Qualitative synthesis*															
Wongpairojpanich 2020	Parallel	Thailand	Light smoker	University	No	NR	Intact	30	2°	AP	AP	Non-resorbable	Non-resorbable	HWch	CBCT and digital tool (direct scanning, STL files with CBCT
Thoma 2020	Parallel	Switzerland	Heavy smoker	Private Practice	Yes	A = yes B = yes C = no	More than 50%	36	2	A = XG B = XG	SH	A = NonCross CM B = NO	NO	KM thickness, CCh	Analogic caliper, master cast digital tool (STL files)
Sapata 2019	Parallel	Brazil	Light smoker	University	Yes	NR	Intact	82	2°	XG	XG	NonCross CM	NonCross CM	KM thickness, CCh, HWch	Master cast and digital tool (STL files)
Fischer 2018	Parallel	Germany	Light smoker	University	No	No	Intact and missing	40	T1 = 1° T2 = 2° T3 = 1° T4 = 2°	XG	SH	T1 = PG T3 = collagen membrane	T2 = NO T4 = NO	CCh	Master cast and digital tool (STL files)
Schnutenhaus 2018	Parallel	Germany	Light smoker	University	Yes	No	More than 50%	60	2°	SH	SH	Collagen membrane	NO	CCh	Master cast and digital tool (STL files)
Tomasi 2018	Split-mouth	Italy	Heavy smoker	NR	Yes	No	Intact	27	2°	XG	SH	NonCross CM	NonCross CM	CCh, VBH, VPH, HWch	Master cast and digital tool (STL files)
Fickl 2017	Parallel	Germany	Light smoker	University	Yes	No	Intact and missing	40	2°	XG	SH	A = PG B = NO C = Cross CM	NO	VBH	Master cast
Zadeh 2016	Split-mouth	Saudi Arabia	Heavy smoker	University	Yes	NR	A, B = intact C, B = missing	36	2°	C = XG E = XG	A = SH B = SH D = SH	Group B: non-resorbable device Group C: non-resorbable device Group E: non-resorbable device	Group A:NO Group D:NO	CCh	Master cast and digital tool (STL files)
Flugge 2015	Split-mouth	Germany	Non-smoker	University	Yes	NR	Intact	38	2°	XG	SH	NO	NO	CCh, VBH	Master cast and digital tool (STL files)
Engler-Hamm 2011	Split-mouth	USA	Non-smoker	University	Yes	Yes	Intact	11	C group = 1° T group = 2°	AG + XG	AG + XG	PLA/PTMC	PLA/PTMC	KM thickness, VBH	Probe and stent
Kesteren 2010	Parallel	USA	Light smoker	NR	Yes	NR	Intact	28	1°	AG	SH	collagen membrane	NO	VBH	Probe and master cast and photo
*Quantitative analysis*															
Vance 2004	Parallel	USA	NR	NR	Yes	Yes	NR	24	2°	AP + AG	XG	CaS barrier	NonCross CM	KM thickness	Ultrasonic gingival meter, stent and photo
Iasella 2003	Parallel	USA	YES	NR	NR	Yes	NR	24	2°	AG	SH	Cross CM	NO	KM thickness	Ultrasonic gingival meter, stent and photo
Ovcharenko 2020	Parallel	USA	NR	University	Yes	NR	Intact	20	2°	AG + XG	AG + XG	NonCross CM	PLA membrane	KM thickness, CCh, HWch	Probe and stent
Clementini 2020	Parallel	Italy	Light smoker	University	Yes	Yes	Intact	30	2°	XG	SH	NonCross CM	NO	KM thickness, VBH, CCh	Probe and master cast, digital tool (STL files with CBCT)
Song 2020	Parallel	Seoul	Heavy smoker	University	Yes	Yes	Intact	40	2°	XG	SH	NonCross CM	NO	KM thickness, VBH	CBCT and digital tool (direct scanning, STL files with CBCT)
Hong 2018	Parallel	USA	YES	University	Yes	Yes	Intact	30	Control = 1° Test = 2°	AG	AG	Cross CM	NonCross CM	KM thickness, VBH	Probe
Natto 2017	Parallel	USA	Light smoker	University	No	Yes	Intact	28	2°	AG	AG	NonCross CM	Collagen sponge	KM thickness, VBH	Probe and stent
Festa 2013	Split-mouth	Italy	Non-smoker	University	No	Yes	Intact	15	2°	XG	SH	cortical porcine laminae	NO	VBH	Probe
Barone 2012	Parallel	Italy	Light smoker	University and Private Practice	Yes	No	Intact	58	C group = 1° T group = 2°	XG	SH	NonCross CM	NO	VBH	Probe
Schneider 2014	Parallel	Switzerland	Heavy smoker	University	Yes	Yes	Intact	40	2°	A = AP; B = XG; C = XG	SH	A = PLGA membrane B = NonCross CM C = PG	D = NO	CCh, HWch	Master cast and digital tool (STL files)
Thalmair 2013	Parallel	Germany	Light smoker	Private Practice	Yes	No	Intact	30	2°	XG	SH	A = PG B = PG C = NO	NO	CCh, HWch	Master cast and digital tool (STL files)

*SH* spontaneous healing; *AU* autogenous bone graft; *XG* xenograft; *AG* allograft; *AP* alloplastic; *NR* not reported; *CMC* carboxymethyl cellulose; *NonCross CM* non-crosslinked collagen membrane; *Cross CM* crosslinked collagen membrane; *PG* autogenous soft tissue punch graft; *ADMG* acellular dermal matrix graft; *PLA* polylactic acid; *PLGA* poly(glycolide-co-lactide) copolymer; *PLA/PTMC* polylactic acid/polytrimethylene carbonate; *HWch* horizontal width change; *CCh* contour change; *VBH* vertical buccal height; *KMT* keratinized mucosa thickness

**Table 2:** Qualitative results

Author Year	Gender (M/F)	Age (mean ± SD or range)		Tooth type/location (Ant/Post)	Arch (max/mand)	Follow-up	Test patients treated	Test patients evaluated	Control patients treated	Control patients evaluated	Complications	Outcome
*Qualitative synthesis*												
		Test	Control									
Wongpairojpanich 2020	7/23	56.07 ± 11	60.73 ± 7.98	13/17	14/16	4 mo	15	15	15	15	No	B-PPM could potentially be used as an alternative choice for ARP Similar outcomes were observed throughout the evaluation period when compared with commercial d-PTFE membrane
Thoma 2020	18/18	A = 53 to 71.5 y B = 55 to 74 y	C = 52 to 71	A = 16/25 B = 5/8	27/9	8 w	A = 12 B = 13	A = 12 B = 13	C = 11	C = 11	No	The thickness of the mucosa in group DBBM-C/CM compared to in group SH, underlines a moderate effect size
Sapata 2019	NR	43.3 ± 10.3 y	41.9 ± 11.9 y	65 A	NR	4 mo	41	33	41	33	No	For main outcome (HWCh) the DBBM group was non-inferior to the DBBM-C group. After 4 months, the use of DBBM was non-inferior to DBBM-C in terms of soft tissue contour changes
Fischer 2018	16/24	55.7 ± 14.85 y		NR	NR	6 mo	T1 = 10 T2 = 10 T3 = 10	T1 = 9 T2 = 8 T3 = 10	10	8	NR	Three technique result in similar buccal contour change, with smallest changes in T1
Schnutenhaus 2018	29/31	24 to 78 y		19/31	NR	6 w	31	31	29	29	NR	In the ARP group, there was a statistically significant smaller reduction of the observed soft tissue contour
Tomasi 2018	11/16	38 to 79 y		28 P	16/12	6 mo	NR	NR	NR	NR	No	In both the test and control groups, the vertical and horizontal dimension was only modestly reduced between baseline and 6 months
Fickl 2017	16/24	55.7 ± 14.85 y		NR	NR	6 mo	NR	NR	NR	NR	No	A and C resulted in significantly less buccolingual dimension loss
Zadeh 2016	NR	NR	NR	13/48P	31/30	6 mo	A = 12 t B = 11 t C = 14 t E = 10 t	A = 12 t B = 11 t C = 14 t E = 10 t	D = 14 t	D = 14 t	NR	SocketKAP, with or without ABBM, significantly limited post-extraction ridge contour loss in intact sockets
Flugge 2015	13/25	28 to 78 y		39/40	49/30	12 w	40 teeth	40 t	39 t	39 t	NR	There was a significant difference of the mean dimensional changes: non-augmented sites showing more resorption than augmented sites
Engler-Hamm 2011	4/7	41.09 ± 14.07 y		24 P	NR	6 mo	11 t	11 t	11 t	11 t	No	MGJ was statistically significantly more coronally displaced in the control group than it was in the test group. Ridge preservation without flap advancement was shown to preserve the buccal keratinized tissue significantly better
Kesteren 2010	NR	NR	NR	9 /17	21/5	6 mo	14	13	14	11 (13 t)	1 implant failed	Midbuccal soft tissue margin position shows no significant difference. The same was for interproximal tissue
*Quantitative analysis*												
Vance 2004	9/15	56 ± 14 y		4/20	19/5	4 mo	12	12	12	12	NR	Soft tissue thickness not significant difference
Iasella 2003	10/14	51.5 ± 13.6 y		25/23	18/6	6 mo	12	12	12	12	NR	Sites in the RP group lost a slight amount of overlying soft tissue thickness, while those in the EXT group gained about 0.5 mm
Ovcharenko 2020	4/16	61 ± 10 y	48 ± 14 y	10/10	16/4	4 mo	10	10	10	10	No	Both the PLA and ADMG groups ha significant gain in soft tissue thickness. At 5 mm apical to the crest, PLA group’s gain was significantly greater than ADMG
Clementini 2020	14/16	A = 55.5 ± 11.6 B = 52.5 ± 7.5	C = 50.5 ± 12.2	16/14	22/8	4 mo	20	20	10	10	NR	No differences were observed in horizontal changes between the two test treatments and spontaneous healing sites. This lack of difference is related to a significant increase in soft tissue thickness in spontaneous healing sites
Song 2020	24/11	55.3 ± 8.33 y	50.8 ± 12.6 y	35 P	NR	6 mo	20	19	20	16	Partial exposure of the bone graft material	The thickness of the mucosa was significantly thinner in ARP group. MGJ moved slightly apically in ARP group and shifted coronally in SH group
Hong 2018	10/20	52.30 ± 17.3 y	48.50 ± 5.0	NR	NR	6 mo	15	14	15	14	No	The width of keratinized tissue, the E group gain’s was greater than C group. Same result has found for keratinized thickness
Natto 2017	17/11	25 to 80 y	30 to 74 y	11/17	23/5	4 mo	14	14	14	14	NR	Differences between the two groups were not statistically significant for all clinical soft tissue measurement variables
Festa 2013	12/18	28 to 58 y	28 to 58 y	NR	NR	6 mo	15 t	15 t	15 t	15 t	No	Both treatments equally preserved the baseline level of the free gingival margin at the neighboring teeth after the extractions
Barone 2012	NR	41.8 ± 14.0 y	39.3 ± 15.5 y	58 P	NR	4 mo	29	29	29	29	NR	Width of keratinized gingiva was better preserved in the test group compared to the control group
Schneider 2014	NR	NR	NR	17/23	NR	6 mo	A = 10 B = 10 C = 10	A = 9 B = 9 C = 10	D = 10	D = 9	NR	Application of DBBM-C/CMor DBBM-C/PG reduced the amount of volume resorption compared to ß-TCP or spontaneous healing without reaching statistically significant difference
Thalmair 2013	18/12	24 to 72 y		A = 4/4 B = 4/4 C = 2/5	24/6	4 mo	A = 8 B = 8 C = 7	A = 8 B = 8 C = 7	D = 7	D = 7	No	Significant differences in dimensional change between the test groups A and B compared with control group D. A significant influence of the soft tissue socket seal leading to a lower degree in shrinkage. The influence of the filler was estimated to be not significant

**Table 3:** Quantitative changes. The mean difference data (baseline-last follow-up)

	Code material test	Code material control	Code seal test	Code seal control	KM change (mm)				Buccal change (mm)				Horizontal change (mm)			
					Test		Control		Test		Control		Test		Control	
					Mean	SD	Mean	SD	Mean	SD	Mean	SD	Mean	SD	mean	SD
Ovcharenko 2020	AG + XG	AG + XG	NonCross	Resorbable synthetic	1.35	1.20	1.1	1.01	-	-	-	-	A = − 1.65 B = − 1.70	A = 0.27 B = 0.34	− 2.86	0.31
Clementini 2020	XG	SH	Non-cross	NO	A = − 0.23 B = − 0.35	A = 0.69 B = 0.71	0.42	0.66	-	-	-	-	− 2.48	0.28	− 2.86	0.32
Song 2020	XG	SH	NonCross	NO	2.17	0.54	3.33	0.99	0.63	1.21	− 0.29	0.60	-	-	-	-
Hong 2018	AG	AG	Cross	NonCross	0.46	0.22	− 0.15	0.23	0.43	0.42	− 1.57	0.51	-	-	-	-
Natto 2017	AG	AG	NonCross	ColS	0.47	1.26	0.07	1.26	-0.08	0.54	− 0.08	1.24	-	-	-	-
Schneider 2014	A = AP; B = XG; C = XG	SH	A = Resorbable synthetic B = CM C = PG	SH	-	-	-	-	-	-	-	-	A = − 1.69 B = − 1.15 C = − 1.16	A = 0.74 B = 0.50 C = 0.68	D = − 1.78	D = 0.82
Festa 2013	XG	SH	Cross	SH					0.1	0.2	0.1	0.2				
Thalmair 2013	XG	SH	A = PG B = PG C = NO	SH	-	-	-	-	-	-	-	-	A = 0.79 B = 0.85 C = 1.45	A = 0.5 B = 0.6 C = 0.7	D = 2.29	D = 1.1
Barone 2012	XG	SH	NonCross	SH	-	-	-	-	1.14	0.8	0.73	0.8	-	-	-	-
Vance 2004	AP + AG	XG	Resorbable synthetic	NonCross	0	0.65	0.1	1.17	-	-	-	-	-	-	–	-
Iasella 2003	AG	SH	Cross	SH	− 0.35	1.11	0.45	1.14	-	-	-	-	-	-	-	-

*NR* not reported, *y* years, *mo* months, *w* weeks

### Qualitative synthesis

Overall, in articles included in the systematic review, 792 surgical sites (454 in the test group and 338 in control groups) were treated, and 759 surgical sites were evaluated (438 in the test group and 321 in control groups). The total number of included patients was 767; however, four included studies [18, 25, 27, 36] reported only the number of teeth, and two others [37, 42] did not report the total number of included patients/teeth.

The smoking status was collected; studies that included smokers of more than 10 cigarettes were considered heavy smokers. Eleven studies include light smoking patients and 5 studies include heavy smokers. Two studies considered smoking patients without specifying frequency and the other 2 studies did not report the information. The presence of a buccal wall was reported in 16 studies (Table 1), two of which [34, 35] specified a threshold of 50% buccal bone height as a criterion for participant inclusion. Additionally, three [28, 37, 42] studies included both sockets intact and compromised buccal walls. A total of 16 studies employed healing by secondary intention, two studies obtained primary healing, and four studies employed a mixture of primary and secondary intention healing. Among the included studies, five studies had samples without molars, three studies had a high proportion of molars in the sample, and one study only included molars. For 11 studies, the follow-up period was 6 months, while four studies reported a follow-up of less than 6 weeks (Table 2). All included studies investigated at least one of the following soft tissue changes post-extraction: KMT, horizontal/vertical soft tissue changes, or 3-dimensional contour changes. It is important to note that out of all the measurements, only KMT always referred to soft tissue changes exclusively. The other indices, especially when measured through STL file superimposition without a CBCT, were a composite measure of both hard and soft tissue dimensional changes. Considerable interstudy methodological heterogeneity was noted regarding the technique for assessing dimensional changes, chosen reference points, studied outcomes, analysis of buccal wall integrity, and also statistical reporting approach (choice to report SD or SE). The graft materials employed in the test groups are listed in order of frequency: fourteen studies used xenograft (XG), four studies used allograft (AG), two studies used a combination of xenograft and allograft (XG+AG), and one study used alloplastic (AP) graft alone as well as in combination with allograft (AP+AG). In the control group, fifteen studies did not use a graft material, two studies employed allograft, two others used xenograft, and two studies used a combination of allograft and xenograft.

### Risk of bias analysis

**Fig. 2:**
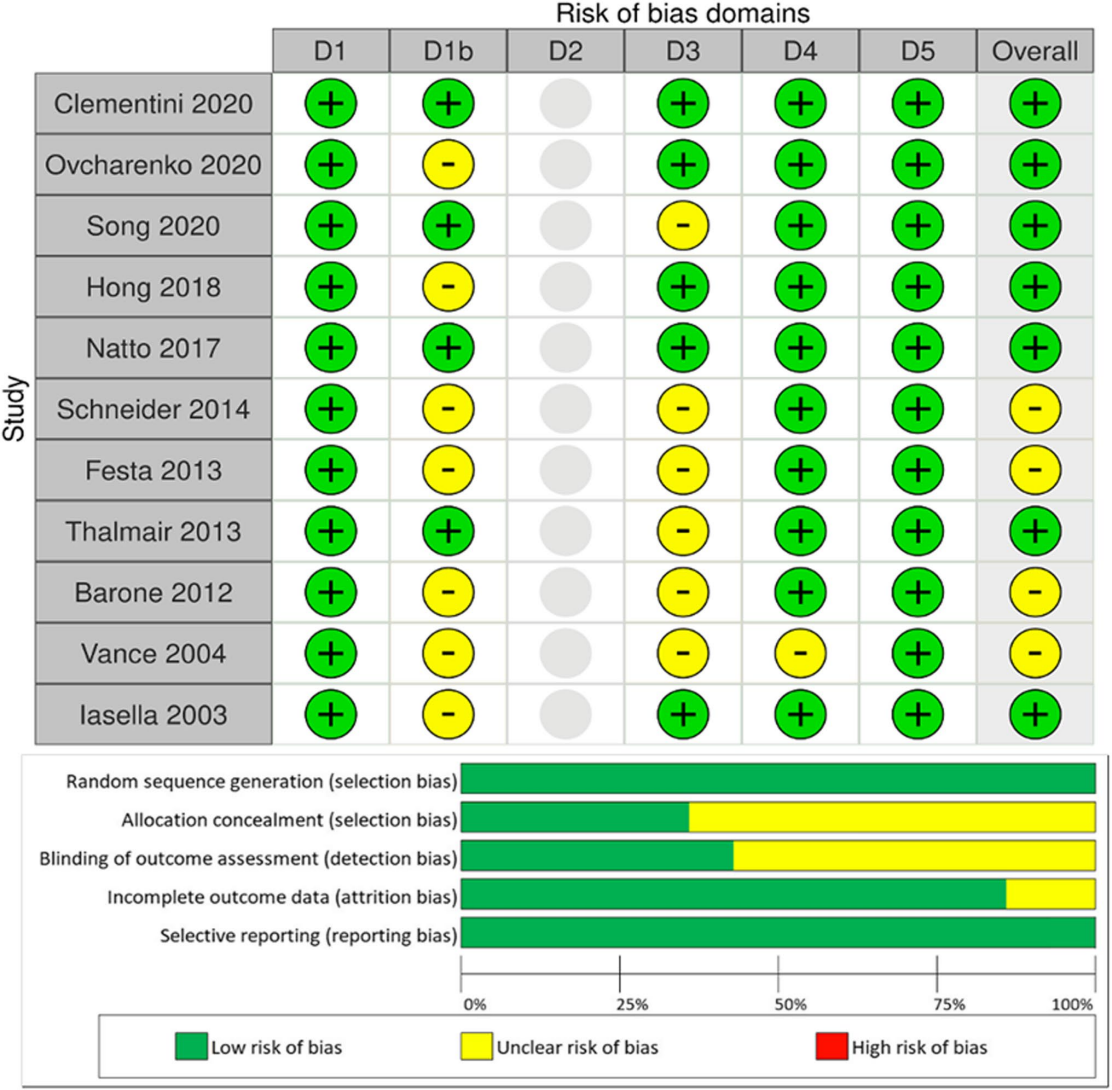
Overall risk of bias plot

Figure 2 shows the results of the risk of bias assessment, which has been performed only for studies included in the quantitative NMA. Of the studies considered for NMA, seven [24, 29, 31, 38, 39, 41, 43] were associated with a low risk of bias, four were associated with a moderate risk [25, 26, 33, 40], and none were associated with a high risk.

### Network meta-analysis

Studies comparing different socket sealing biomaterials were considered during the NMA. Overall, it was impossible to assess the impact of healing type (primary/secondary intention) on soft tissue dimensional changes, as this variable was heterogeneously distributed and could not be evaluated. Of the studies in the NMA, eight included smoking patients (24, 26, 29, 33, 38, 39, 41, 43), of which 4 (24, 26, 39, 43) were only light smokers and 2 (33, 38) were also heavy smokers. Two studies (31, 40) do not report the smoking status and one (25) considers strictly non-smoking patients.

## Keratinized mucosa thickness changes

**Fig. 3:**
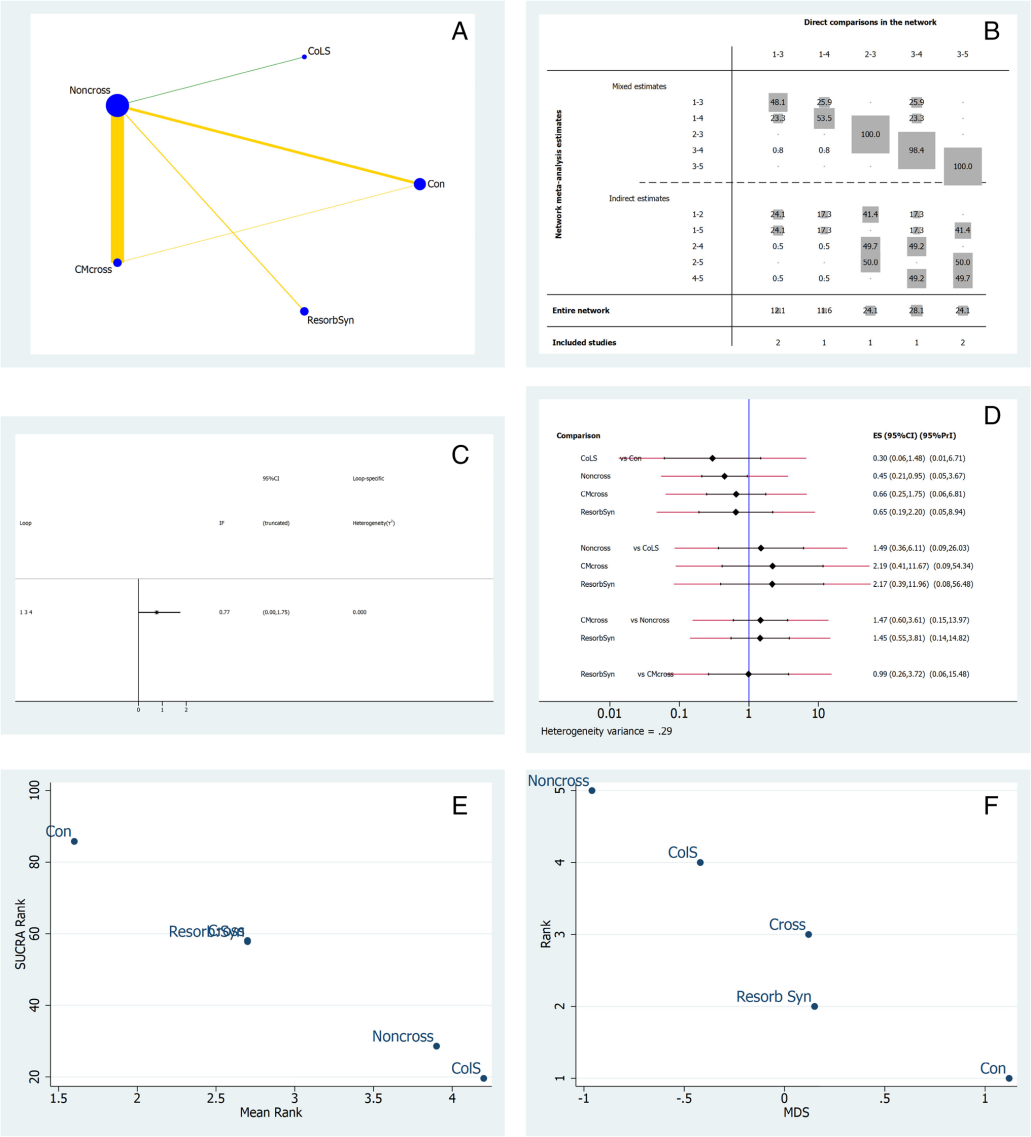
NMA for keratinized mucosa thickness. The size of the circle (Node-blue) is proportional to the number of subjects randomized to that treatment. The thickness of the lines is proportional to the number of studies investigating each comparison (***A***). In NMA, identifying comparisons with large and small contribution is of interest. Therefore, contribution plots are developed to identify the flow of direct, indirect, and mixed evidence in the network (***B***). Direct comparisons 2 vs 3 and 5 vs 3 contributed most to the evidence in the network. Inconsistency plots are used to rule out statistical inconsistency and validate the network (***C***). The predictive interval plot is the interval within which the estimate of a future study is expected to lie. There are three lines, i.e., redline, blue line at the center, black line. The black line is representative of confidence interval (CrI), the red line illustrates the predictive interval (Prl), and the central blue line is the line of no effect. Only in this case, the plot must be read in opposite way as a large value of KMT represents ARP failure (***D***). The multidimensional scale ranking ranks different treatments with their relative incoherence or ranks according to their dissimilarity (***E***). The surface under the cumulative ranking curve (SUCRA) is a numeric presentation of the overall ranking and presents a single number associated with each treatment. SUCRA values range from 0 to 100% (0 to 1). The higher the SUCRA value and the closer to 100%, the higher the likelihood that a therapy is in the top rank (***F***). **A** Network geometry plot, **B** contribution plot, **C** inconsistency plot of entire network, **D** predictive interval and confidence interval plot, **E** surface under the cumulative ranking curve (SUCRA), **F** multidimensional scale ranking (MDS) for keratinized mucosa thickness, Con (treatment n.1) = control; ColS (n.2) = collagen sponge; CM-NonCross (n.3) = collagen membrane non-crosslinked; CM-Cross (n.4) = collagen membrane crosslinked; Resorb Syn (n.5) = resorbable synthetic

Seven studies reporting thickness measurements were included in the NMA [25, 29, 31, 38, 40, 41, 43]. Figure 3A illustrates the network geometry plot for KMT outcomes after ARP. The colored edges represent the level of bias in the majority of trials, weighted according to the number of studies in each comparison. The most common comparison was between non-crosslinked collagen membranes (CM-NonCross) and crosslinked collagen membranes (CM-Cross). The risk of bias was low for CM-NonCross and collagen sponge (ColS) comparisons (green line), and moderate between other comparisons (yellow lines). In the contribution plot (Fig. 3B), the majority of the evidence is derived from the CM-NonCross versus CM-cross (28.1%) comparison, followed by CM-NonCross vs ColS and resorbable synthetic comparisons (both at 24.1%). In the inconsistency plot (Fig. 3C), there were no statistically significant inconsistencies in the loop formed by the control, CM-Cross, and CM-NonCross groups. This is suggestive of differences between the direct and indirect effect estimates for the same comparisons. Figure 3D illustrates the predictive interval and confidence interval plots. The CM-NonCross group exhibited a favorable effect estimate, and CM-Cross was likely to achieve worse results than CM-NonCross in a direct comparison. The ColS group was most likely to perform better in future clinical studies. A resorbable synthetic membrane was likely to achieve worse results compared to CM-NonCross and its effect size was comparable to CM-Cross and similar to the control group. The treatments were ranked for performance based on KMT utilizing surface under the cumulative ranking curves (SUCRA) measurements [45, 46]. The control treatment group was ranked lowest, while ColS was ranked highest followed by CM-NonCross (Fig. 3E). Multidimensional scale ranking (MDS) (Fig. 3F) showed that the control group, resorbable synthetic, and CM-Cross membranes were positioned after the 0 line displaying how these interventions are more similar to the control group than to CM-NonCross [47], in agreement with SUCRA rank.

## Vertical buccal height

**Fig. 4:**
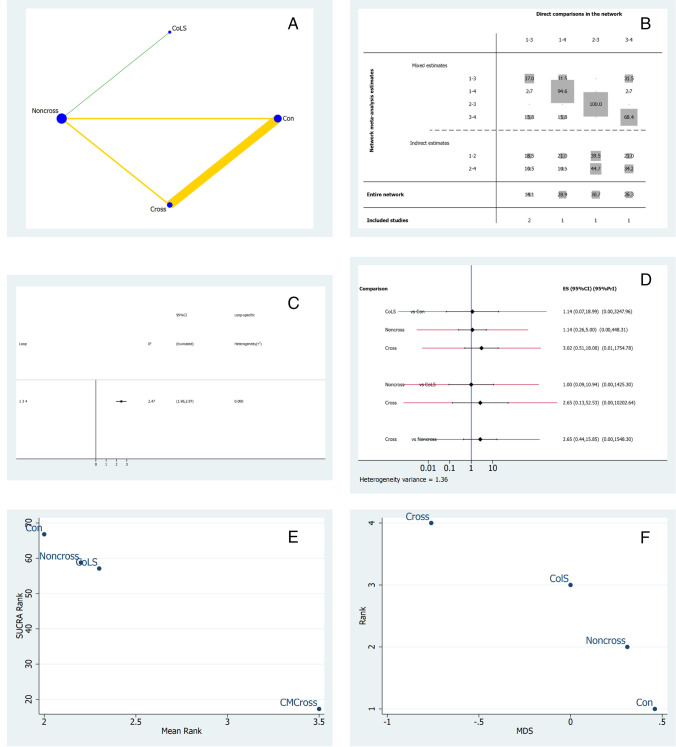
NMA for vertical buccal height*.* **A** Network geometry plot, the color of the edges corresponds to the average bias risk and the size of the blue dots is proportional to the sample size of that study group. The thickness of the lines demonstrates the number of comparisons made between the two groups of treatment. **B** Contribution plot. **C** Inconsistency plot of entire network. **D** Predictive interval and confidence interval plot: we can consider the values on the left as favoring second intervention and right as favoring first intervention [41]. **E** Surface under the cumulative ranking curve (SUCRA). **F** Multidimensional scale ranking (MDS) for keratinized mucosa thickness. Con (treatment n.1) = control; CM-Noncross (n.2) = collagen membrane non-crosslinked, ColS (n.3) = collagen sponge;; CM-Cross (n.4) = collagen membrane crosslinked

Five studies were included in the NMA regarding vertical keratinized buccal mucosal height changes [24–26, 29, 38]. Figure 4A illustrates the network geometry plot for vertical buccal height outcomes. The most common comparison was between CM-NonCross and the control group. The risk of bias was low between CM-Cross and ColS, and moderate between comparisons with a yellow line. The contribution plot (Fig. 4B) shows that the comparison between ColS and CM-NonCross was given only by direct comparisons (100%), and was the most influential in terms of indirect comparisons and also of the entire network (30.7%). The comparison between the control group and CM-Cross was mainly formed by direct comparisons (94.6%), and was the second most influential in the entire network (28.9%). Figure 4C represents the inconsistency plot; the loop formed between the control, CM-NonCross, and CM-Cross groups had statistically significant inconsistencies (*p* > 1.81). Figure 4D shows the predictive interval and confidence interval plots. The results of the predictive interval plot do not show significant differences, although ColS is likely to perform better compared to CM-NonCross in future clinical trials. According to the SUCRA ranking, CM-Cross was ranked highest followed by ColS (Fig. 4E). MDS (Fig. 4F) demonstrated coherence with SUCRA rank; the CM-Cross group was very distant from the other study groups and ColS did not cross the 0 line, so the difference between CM-Cross and other interventions is remarkable.

## Horizontal width changes

**Fig. 5:**
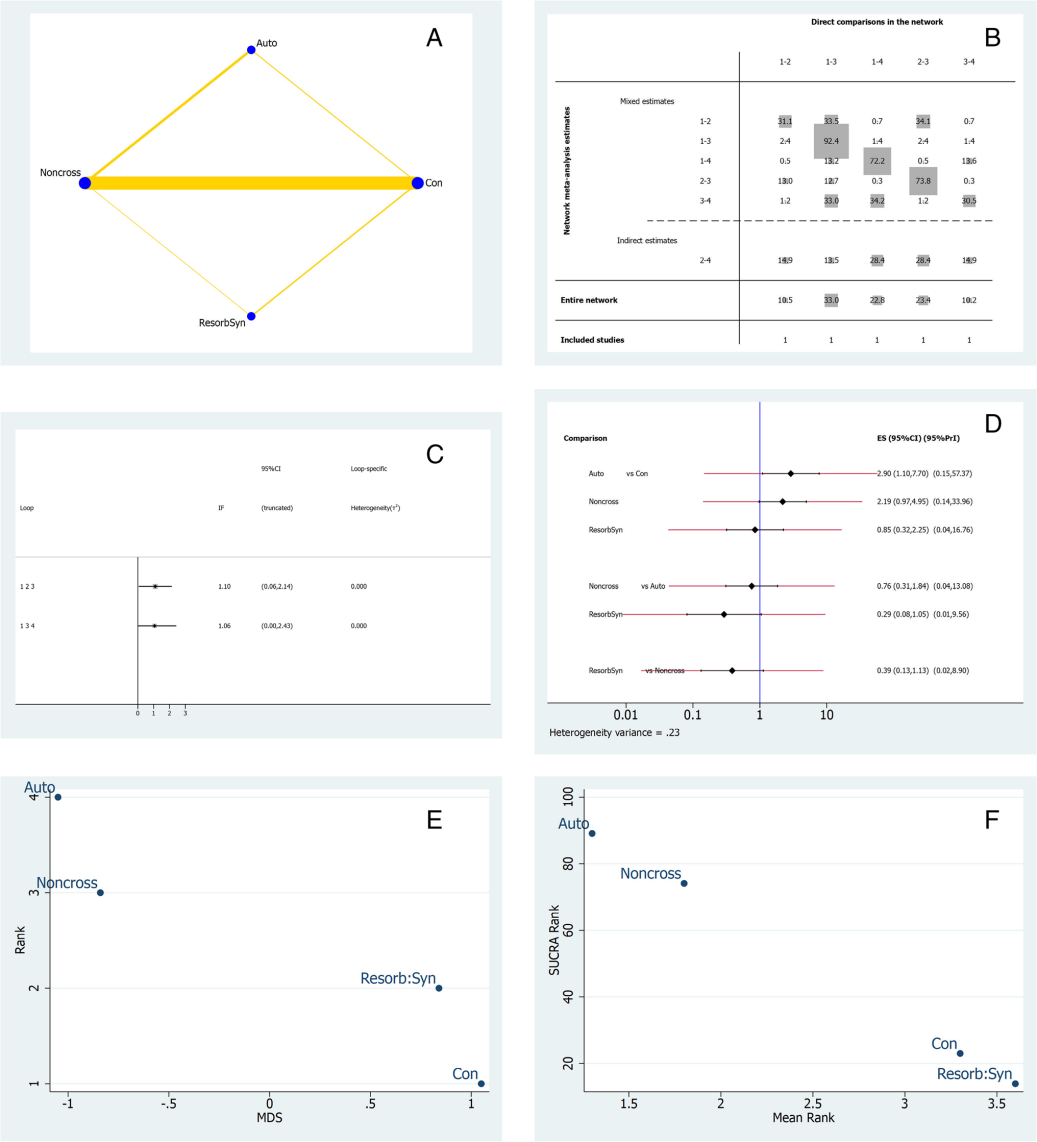
NMA for horizontal changes. **A** Network geometry plot, **B** contribution plot, **C** inconsistency plot of entire of entire network, **D** predictive interval and confidence interval plot, **E** surface under the cumulative ranking curve (SUCRA), **F** multidimensional scale ranking (MDS) for keratinized mucosa thickness. Con (treatment n.1) = control; Resorb Syn (n.2) = resorbable synthetic; CM-NonCross (n.3) = collagen membrane non-crosslinked; Auto (n.4) = autogenous graft punch

In regards to three-dimensional soft tissue contour changes, the horizontal linear changes reported by four studies were considered for NMA [31, 33, 39, 43]. Figure 5A shows the network geometry plot. The most common comparison with the largest sample size was between the control and CM-NonCross group. The contribution plot (Fig. 5B) showed that the control versus CM-NonCross group was formed mainly by direct comparisons (92.4%), and that this comparison was the most influential in the entire network. The indirect estimates were formed by autogenous versus control group (28.4%), as well as resorbable synthetic versus CM-NonCross (28.4%) groups. The risk of bias was moderate between all three comparisons. Figure 5C represents the inconsistency plot and demonstrated insignificant inconsistency. In the predictive interval and confidence interval plots (Fig. 5D), the autogenous and CM-NonCross group performed statistically significantly (*p* < 0.001) better than the control group. The autogenous soft tissue group ranked highest in the SUCRA ranking (Fig. 5E) followed by the CM-NonCross group. In the MDS, the autologous soft tissue graft and CM-NonCross groups ranked superiorly (Fig. 5F). Thalmair et al. and Schneider et al. were the only two studies identified with data related to PG in the horizontal outcome and there were no other studies that considered autogenous soft tissue grafts in the comparative group.

## Discussion

The findings of the present systematic review and network meta-analysis clarified that hard and soft tissues behave differently after alveolar ridge preservation as a response to the choice of the biomaterials used to seal the socket. These results are in line with those reported by the other systematic review on this topic [14]. Despite the small number of studies investigating soft tissue outcomes, MacBeth et al. confirm how GBR procedures without achieving primary closure provide an increase in soft tissue width and a slight decrease in thickness compared to no intervention group.

In our review, keratinized mucosa thickness was considered the most important parameter in assessing soft tissue regeneration. It must be noted that vertical and horizontal changes of soft tissues are difficult to analyze without the hard tissue component.

NMA is a useful approach for comparing multiple treatment arms, where the evidence is drawn from both direct and indirect comparisons. In this way, NMA facilitates indirect comparison of interventions for which direct comparisons have not yet been carried out in the literature. In the present study, the included treatment groups were selected based only on the biomaterial used for the sealing technique regardless of the used bone filler. Although substantial heterogeneity was present among included studies for the type of used bone filler, there is no robust evidence indicating that the type of graft material may directly affect soft tissue dimensional changes [48–50].

An important finding was that KMT represented the most homogeneous outcome. The findings on mucosal thickness changes were in agreement that spontaneous healing leads to increased bone resorption but greater soft tissue thickness [38]. However, it needs to be kept in mind that the surgical technique may have also influenced this result. Hong et al. reported that full thickness flap elevation followed by application of non-crosslinked membranes and primary healing resulted in reduced soft tissue dimensions compared to the use of crosslinked membranes applied during a minimally invasive surgical technique and secondary healing [29]. This important aspect needs to be underlined in the light of the finding that the direct comparison between crosslinked and non-crosslinked membranes was the most influential of the entire KMT network. However, in line with NMA outcomes, crosslinked collagen membranes ranked highest, followed by synthetic resorbable membranes which also performed better than non-crosslinked collagen membranes. The direct evidence was, to the greatest extent, due to the use of these materials, and no statistically significant inconsistencies were observed (Fig. 3B). Whether given of the result obtained, we have to underline that in the analyzed sample, the number of molar teeth is double of the anterior ones. Furthermore, suture technique and incision design play also important roles during the healing phase. Positive results have been observed when flaps are not elevated and the membrane is left intentionally exposed [27, 29]. Also, these findings could be influenced by the fact that posterior teeth will generate more soft tissue by secondary intention than the anterior teeth due to the larger surface area of the socket opening. However, this approach does not apply to all clinical situations and can lead to increased microbial-related complications. Despite this, only two complications were reported in the included articles (Table 2), although ten included articles reported post-operative antibiotic administration in their protocols.

Positive effects regarding vertical buccal height were reported in six studies. Several measurement methods were adopted to evaluate VBH: some studies reported linear measurements [24, 26, 29, 42], mucogingival junction shift [27, 38], recession with respect to neighboring teeth [25, 30], or volumetric/contour changes [37]. Interestingly, many authors stated that soft tissue management is crucial for maintaining keratinized mucosa height [27, 28, 38, 51]. At the same time, the presence of the graft material does not seem to influence this outcome. On the other hand, the most relevant factor influencing the amount of newly formed soft tissue seems to be related to the sealing procedure. A moderate level of evidence favors the use of soft tissue punch autografts and collagen membranes.

From a clinical point of view, the data indicate that crosslinked membranes can be considered, but one has to remember that the loop formed between control, CM-NonCross, and CM-Cross groups has statistically significant inconsistencies, and the overall risk of bias of the specific network was moderate. Non-crosslinked collagen membranes revealed no statistically significant differences compared to the use of collagen sponges. Again, the size of the effect for the CM-NonCross group may have been influenced by the different healing types (primary/secondary intention) utilized in Hong et al. and Barone et al. [26]. Although the results suggest that sealing the socket with a collagen sponge may increase the vertical keratinized soft tissue height, the MDS (Fig. 4F) showed how ColS treatment group are closer to CM-NonCross and control than to CM-Cross. However, conclusive clinical recommendations from the present NMA should be taken with caution also because the sample is not homogeneous about tooth location so the results obtained are more relevant for posterior teeth. This finding can be explained by the fact that papilla height is very closely related to the underlying bone levels and its preservation [52]. Different socket sealing materials may result in alterations in vertical bone resorption, thus affecting the vertical height of keratinized mucosa.

The third outcome, concerning the three-dimensional contour changes and horizontal modifications, was very challenging to investigate because most of the articles included in this NMA assessed soft tissue profile variations by overlapping STL files. STL files were obtained through indirect or direct methods, both of which are associated with a risk of errors. For example, indirect methods utilizing physical impression materials may compress the tissues, resulting in undersized models, while direct scanning may not work correctly with the presence of blood or moisture.

For the NMA, only horizontal linear measurements were considered. For this reason, only four studies were included. The evidence from all interventions for horizontal outcomes was well distributed, although CM-NonCross and control groups represented the most influential comparison in the network (92.4%). The SUCRA plot showed that autogenous grafts and non-crosslinked collagen membranes ranked highest, and the risk of bias was mainly moderate. It is important to note that the data related to punch graft (PG) is scarce both in KMT or VBH, and only two studies (33, 39) were identified with data related to PG in the horizontal outcome. Therefore, there is limited evidence on the effect of autogenous grafts on soft tissue outcomes. Despite this, no statistically significant inconsistencies were found between loops, and the quality of evidence for this outcome was moderate. This is in agreement with the qualitative analysis which showed that primary closure through autogenous soft tissue grafts seemed to be the most efficient technique for preserving horizontal dimensions [28, 33, 39. 42], although harvesting palatal tissue creates additional discomfort for the patient which is an important clinical limitation. The addition of non-crosslinked membranes led to better results compared with resorbable synthetic membranes. In addition, healing by secondary intention (i.e., leaving the membranes intentionally exposed) is associated with a faster absorption rate relative to healing by primary intention. Clinical effects such as tissue dimensional changes are halved using DBBM compared to spontaneous healing, regardless of the socket sealing material chosen [39, 42]. However, other studies failed to report such a marked influence of biomaterial grafts on three-dimensional changes [37, 39]. Horizontal ridge changes differ depending on the choice of bone filler biomaterial: DBBM may have an advantage over other materials due to its slow degradation rate, but the addition of collagen (DBBM-C) does not seem to confer additional benefits [32, 33].

Many previous studies have found a correlation between thick buccal bone plates and lower resorption rates, such that in sites with thick buccal walls, the benefits of ARP may be less evident [36]. This was confirmed by Clementini et al., where the buccal wall was mostly greater than 1 mm thick, and differences in soft tissue dimensions were not found [42]. Additionally, when bone resorption is severe, soft tissues may experience thick growth to compensate, and vice versa when bone resorption is mild [37, 51].

It should be noted that volumetric measurements do not allow distinction between hard and soft tissues changes. These alterations reflect a combination of horizontal and vertical changes, such that linear measurements alone may not accurately reflect the true clinical scenario. As reported by Sanz-Martin, measurements obtained by overlapping DICOM and STL files have a high correlation with histological linear measurements. In that study, the difference between micro-CT and STL measurements was always between 0.05 and 0.07 mm with Lin’s concordance correlation coefficient between 0.80 and 0.90 [53]. Three included studies [38, 43, 44] superimposed STL files on CBCT files to monitor soft tissue dimensional changes, which is likely a better way to isolate the soft tissue dimensional changes as opposed to just utilizing STL files alone which do not allow for an accurate analysis of soft tissue dimensional changes.

Ultimately, soft tissue dimensions play an important role in implant site development and implant therapeutic outcomes [54], and proactive management of the extraction socket through ARP is an important early step in this process. KMT, vertical soft tissue height, and 3D contour seem to be influenced by different variables. The results of the present study suggest that ARP is capable of mitigating the extent of soft tissue dimensional changes post-extraction. There is moderate evidence suggesting that crosslinked collagen membranes and autogenous soft tissue grafts are effective in terms of maintaining soft tissue dimensions post-extraction. While collagen sponges are likely to perform better in future studies, this biomaterial choice needs more clinical evidence to substantiate its use (Table 4, Table 5, and Table 6). Since the level of bias of the overall network was moderate, more clinical trials directly comparing crosslinked collagen membranes, non-crosslinked collagen membranes, and collagen sponges with less methodological heterogeneity regarding the surgical technique (i.e., flap elevation) and type of healing are needed to strengthen the state of the evidence.

**Table 4:** Quality of direct, indirect, and network evidence for horizontal outcome

Outcomes	Comparison	Direct evidence		Indirect evidence		Network meta-analysis	
		Odds ratio (95% CI)	Quality of evidence	Odds ratio (95% CI)	Quality of evidence	Odds ratio (95% CI)	Quality of evidence
Horizontal width change (HWch)	Auto vs Con (2 vs 1)	1.47 (0.22, 2.71)	Moderate	0.58 (− 0.81, 1.97)	Moderate	0.88 (− 0.98, 2.75)	Moderate
	CM-NonCross vs Con (3 vs 1)	0.37 (0.14, 0.61)	Low	1.46 (0.65, 2.27)	Moderate	− 1.08 (− 1.93, − 0.24)	Moderate
	Resorb:Syn vs Con (5 vs 1)	0.08 (− 1.21, 1.39)	Low	− 0.67 (− 2.53, 1.17)	Moderate	0.76 (− 1.50, 3.03)	Moderate
	CM-NonCross vs Auto (3 vs 2)	0.01 (− 1.03, 1.05)	Low	− 0.87 (− 2.42, 0.67)	Moderate	0.88 (− 0.98, 2.75)	Moderate
	Resorb:Syn vs Auto (5 vs 2)	-	-	-	-	-	-
	Resorb:Syn vs CM-NonCross (5 vs 3)	− 1.34 (− 2.92, 0.22)	Moderate	− 0.58 (− 2.21, 1.04)	Moderate	-0.76(-3.03,1.50)	Moderate

High quality (⊕ ⊕ ⊕)—we are very confident that the true effect lies close to that of the estimate of the effect. Moderate quality (⊕ ⊕  ⊕ O)—we are moderately confident in the effect estimate: the true effect is likely to be close to the estimate of the effect, but there is a possibility that it is substantially different. Low quality (⊕ ⊕ OO)—our confidence in the effect estimate is limited: the true effect may be substantially different from the estimate of the effect. Very low quality (⊕ OOO)—we have very little confidence in the effect estimate: the true effect is likely to be substantially different from the estimate of effect

**Table 5:** Quality of direct, indirect, and network evidence of keratinized mucosa thickness outcome

Outcomes	Comparison	Direct evidence		Indirect evidence		Network meta-analysis	
		Odds ratio (95% CI)	Quality of evidence	Odds ratio (95% CI)	Quality of evidence	Odds ratio (95% CI)	Quality of evidence
Keratinized mucosa thickness (KMT)	ColS vs Con (2 vs 1)	-	-	-	-	-	-
	Con Vs NonCross (1 vs 3)	− 0.67 (− 1.60, 0.26)	Moderate	− 1.40 (− 3.35, 0.53)	Moderate	0.73 (− 1.41, 2.89)	Moderate
	Con vs Cross (1 vs 4)	− 0.8 (− 2.30, 0.70)	Moderate	− 0.06 (− 1.59, 1.47)	Low	− 0.73 (− 2.89, 1.41)	Moderate
	Con vs Resorb:Syn (1 vs 5)	-	-	-	-	-	-
	NonCross vs ColS (3 vs 2)	-	-	-	-	-	-
	ColS vs Cross (2 vs 4)	-	-	-	-	-	-
	ColS vs Resorb:Syn (2 vs 5)	-	-	-	-	-	-
	Cross vs Non-cross (4 vs 3)	0.61 (− 0.61, 1.83)	Moderate	− 0.12 (− 1.90, 1.64)	Low	0.73 (− 1.41, 2.89)	Moderate
	NonCross vs Resorb:Syn (3 vs 5)	-	-	-	-	-	-
	Resorb:Syn vs Cross (5 vs 4)	-	-	-	-	-	-

High quality (⊕ ⊕  ⊕ ⊕)—we are very confident that the true effect lies close to that of the estimate of the effect. Moderate quality (⊕ ⊕  ⊕ O)—we are moderately confident in the effect estimate: the true effect is likely to be close to the estimate of the effect, but there is a possibility that it is substantially different. Low quality (⊕ ⊕ OO)—our confidence in the effect estimate is limited: the true effect may be substantially different from the estimate of the effect. Very low quality (⊕ OOO)—we have very little confidence in the effect estimate: the true effect is likely to be substantially different from the estimate of effect

**Table 6:** Quality of direct, indirect, and network evidence for buccal outcome

Outcomes	Comparison	Direct evidence		Indirect evidence		Network evidence	
		Odds ratio (95% CI)	Quality of evidence	Odds ratio (95% CI)	Quality of evidence	Odds ratio (95% CI)	Quality of evidence
Vertical Buccal Height (VBH)	Con vs ColS (1 vs 2)	-	-	-	-	-	-
	Con vs NonCross (1 vs 3)	0.61 (− 2.66, − 1.13)	Moderate	− 1.89 (− 2.66, − 1.13)	Moderate	2.51 (1.58, 3.43)	Moderate
	Con vs Cross (1 vs 4)	0.1 (− 0.39, 0.59)	Moderate	2.61 (1.83, 3.38)	Moderate	− 2.51 (− 3.43, − 1.58)	Moderate
	ColS vs NonCross (2 vs 3)	-	-	-	-	-	-
	ColS V Cross	-	-	-	-	-	-
	Cross vs NonCross (4 vs 3)	2.0 (1.41, 2.58)	Moderate	− 0.51 (− 1.22, 0.20)	Low	2.51 (1.58, 3.43)	Moderate

High quality (⊕ ⊕  ⊕ ⊕)—we are very confident that the true effect lies close to that of the estimate of the effect. Moderate quality (⊕ ⊕  ⊕ O)—we are moderately confident in the effect estimate: the true effect is likely to be close to the estimate of the effect, but there is a possibility that it is substantially different. Low quality (⊕ ⊕ OO)—our confidence in the effect estimate is limited: the true effect may be substantially different from the estimate of the effect. Very low quality (⊕ OOO)—we have very little confidence in the effect estimate: the true effect is likely to be substantially different from the estimate of effect

The limitations of this review include heterogeneity in measurement techniques for soft tissue dimensional changes and follow-up durations for included studies. A sufficient follow-up duration of at least 6 months is key, as scientific evidence has demonstrated that the majority of post-extraction tissue changes occur within the first 12 months [14]. In addition, the strict inclusion of only RCTs might have led to the exclusion of clinical articles or gray literature which may have increased the sample size allowing for more powerful analyses. Lastly, the presence of buccal walls, the heterogeneity among the bone fillers used, the different smoking status, and the administration of pre-/post-operative antibiotics were confounding factors that may have also influenced the results. On the other hand, we can state that only 2 [40, 41] studies included in the systematic review reported soft tissue or linear ridge measurement as a secondary outcome. Both studies were included in the NMA.

SUCRA in NMA is a numerical ranking designated to each competing treatment based on their performance. The higher the SUCRA value (close to 100%), the greater the likelihood that the biomaterial is in the top rank, and when the value is close to “0,” it is more likely that the biomaterial is in the lower rank. Sometimes, a biomaterial or therapy is ranked higher for effects, but the adverse events are far worse than with the other materials. In this case, clinicians should be careful in selecting the biomaterials based only on higher SUCRA ranking. Some of the reasons why clinicians have to be careful and consider the following factors are the following:

1. Quality of evidence should be taken into account because insufficient clinical trials for the specific biomaterials would give low certainty or confidence and therefore cannot be trusted.
2. When there are multiple outcomes, the rankings for specific biomaterial vary in different outcomes.
3. Cost and clinicians’ familiarity with the use of specific biomaterial should also be taken into consideration.
4. Some of the biomaterials might have ranked closely, i.e., the ranking difference is less between first and second ranked material.
5. SUCRA may not capture the apparent difference between the biomaterials.

There is also an issue of disconnection when authors try to make their study unique and novel. When there is only one study and there are only such comparisons between biomaterials, there will be a disconnection (the lines in the network plot will not be connected to either control or any other biomaterial). In this case, further analysis like predictive interval, SUCRA ranking, and MDS ranking will not be possible.

Therefore, to draw more definitive clinical conclusions, future studies should focus on better delineating the relationship between soft and hard tissue dimensional changes after ARP; in this regard, the superimposition of STL scans and CBCT could be helpful. Furthermore, paying attention to the difference between molars and non-molars and to the influence of bone filler biomaterials compared to socket sealing materials, we will conclude high clinical relevance. As previously mentioned, the homogeneity of study groups in studies investigating the ARP procedures is really important for this type of meta-analysis.

## Conclusions

Within their limitations, the findings of the present systematic review and NMA confirmed that the use of crosslinked collagen membranes and autogenous soft tissue grafts, with a minimum of 6-week follow-up, represented the best biomaterial choices for sealing sockets during ARP in terms of minimizing post-extraction soft tissue dimensional shrinkage.

## Abbreviations

ARP: Alveolar ridge preservation
AU: Autogenous bone
AG: Allografts
AP: Alloplastic grafts
ATG: Autogenous tooth grafts
Auto: Autogenous soft tissue graft
BIO: Bioactive agents
CrI: Confidence interval
CM-NonCross: Non-crosslinked collagen membranes
CM-Cross: Crosslinked collagen membranes
ColS: Collagen sponge
C-DBBM: Deproteinized bovine bone mineral with 10% collagen
DBBM: Deproteinized bovine bone matrix
HWch: Horizontal width changes
IRR: Interrater reliability
KMT: Keratinized mucosa thickness
MA: Bone marrow aspirates
MDS: Multidimensional scale ranking
NMA: Network meta-analysis
PG: Autogenous soft tissue punch
PrI: Prediction interval
ResorbSyn: Resorbable synthetic membranes
SD: Standard deviation
SE: Standard error
SUCRA: Surface under the cumulative ranking curve
VBH: Vertical buccal height
XG: Xenograft
